# A pattern-discovery-based outcome predictive tool integrated with clinical data repository: design and a case study on contrast related acute kidney injury

**DOI:** 10.1186/s12911-022-01841-6

**Published:** 2022-04-15

**Authors:** Yuxi Li, Tak-Ming Chan, Jinghan Feng, Liang Tao, Jie Jiang, Bo Zheng, Yong Huo, Jianping Li

**Affiliations:** 1 Department of Cardiology, Peking University First Hospital, 8 Xishiku St., Xicheng District, Beijing, 100034 China; 2 Digital Innovation Department, WuXi AppTec, Shanghai, China; 3 BI X Digital Lab, Boehringer Ingelheim, Shanghai, China; 4 Big Data and AI of Philips Research China, Shanghai, China

**Keywords:** Machine learning, Predictive tool, Pattern discovery, Acute kidney injury

## Abstract

**Background:**

Clinical data repositories (CDR) including electronic health record (EHR) data have great potential for outcome prediction and risk modeling. We built a prediction tool integrated with CDR based on pattern discovery and demonstrated a case study on contrast related acute kidney injury (AKI).

**Methods:**

Patients undergoing cardiac catheterization from January 2015 to April 2017 were included. AKI was identified based on Acute Kidney Injury Network definition. Predictive model including 16 variables covered in existing AKI models was built. A visual analytics tool based on pattern discovery was trained on 70% data up to August 2016 with three interactive knowledge incorporation modes to develop 3 models: (1) pure data-driven, (2) domain knowledge, and (3) clinician-interactive, which were tested and compared on 30% consecutive cases dated afterwards.

**Results:**

Among 2560 patients in the final dataset, 189 (7.3%) had AKI. We measured 4 existing models, whose areas under curves (AUCs) of receiver operating characteristics curve for the test dataset were 0.70 (Mehran's), 0.72 (Chen's), 0.67 (Gao's) and 0.62 (AGEF), respectively. A pure data-driven machine learning method achieves AUC of 0.72 (Easy Ensemble). The AUCs of our 3 models are 0.77, 0.80, 0.82, respectively, with the last being top where physician knowledge is incorporated.

**Conclusions:**

We developed a novel pattern-discovery-based outcome prediction tool integrated with CDR and purely using EHR data. On the case of predicting contrast related AKI, the tool showed user-friendliness by physicians, and demonstrated a competitive performance in comparison with the state-of-the-art models.

## Background

Clinical data repositories (CDRs) covering Cardiovascular Information Systems (CVIS) [1] and electronic health records (EHR) have great potential for outcome prediction and risk modeling. However, most CDRs were only used for data displaying, and using data from CDR for outcome prediction often requires careful study design and sophisticated modeling techniques before a hypothesis can be tested. Without requiring careful and sophisticated study design, predictive models of machine learning fitted from population-specific historical CDR records (training data) show great value in healthcare applications [2]. However, they are often not easy to follow by doctors, and challenge exists in predicting real-world unseen cases (testing data), which often show changed distributions of the outcome target in a way not foreseen by training data. This challenge, called concept drift [3], could not be easily addressed in machine learning with training–testing split settings. We argue that incorporating clinical domain knowledge in an intuitive way could improve predictive models against concept drift.

Contrast related acute kidney injury (AKI) is among the most common complications induced by use of contrast [4, 5]. It is strongly associated with late renal and cardiovascular adverse events. While established AKI risk models exist [5–7], they were found to be less predictive compared to models fitted from a different population [8–10]. The prevalence of AKI varies and might be changed with associated change of contrast dosage in procedures, introducing concept drift challenge for predictive models fitted from training data. To bridge the above gap, a prediction tool integrated with CDR based on pattern discovery was built and in this case study, we focus on AKI after cardiac catheterization.

## Methods

As previous described [11], patient records undergoing cardiac catheterization and percutaneous coronary intervention (PCI) from January 13, 2015 to April 27, 2017 in Peking University First Hospital were included, a cardiovascular CDR integrated with multiple hospital informatics systems was established to provide the foundation with retrospective structured data registries. The following exclusion criteria was used: dialysis, end-stage renal disease, renal transplant, or missing pre- or post-procedural creatinine data. To prevent the potential missing data, structured prior medical history and vital signs was entered by residents through a composer tool integrated with the EHR admission note system. Crucial data such as left ventricular ejective fraction (LVEF) was extracted from structured echocardiogram reports. A total of 16 pre-operative and in-operative variables covered in representative existing AKI models including Mehran’s score [5], Chen’s score [8], Gao’s score [9], and Age, Glomerular filtration rate and Ejection Fraction (AGEF) score [6] were used for predictive models. We refrained from introducing extra variables here to stay focused on how intuitive domain knowledge incorporation, instead of mixing contribution from extra information, could improve predictive modeling for AKI. The Institutional Review Board at Peking University First Hospital approved this study, and all data was de-identified and informed consent was waived for the retrospective data.

AKI was identified based on Acute Kidney Injury Network (AKIN) definition, which was increase of serum creatinine (≥ 0.3 mg/dL increase, or 1.5-fold or more increase) from most recent baseline before the procedure to the post-procedure 7-day peak [4], and the urine output criterion for AKI diagnosis was not considered in this study. Based on previous studies, AKI is a typical imbalanced target in predictive modeling like many outcomes in clinical practice. Furthermore, recent patients tend to have a lower rate of AKI in the whole cohort which is potentially a concept drift.

**Fig. 1:**
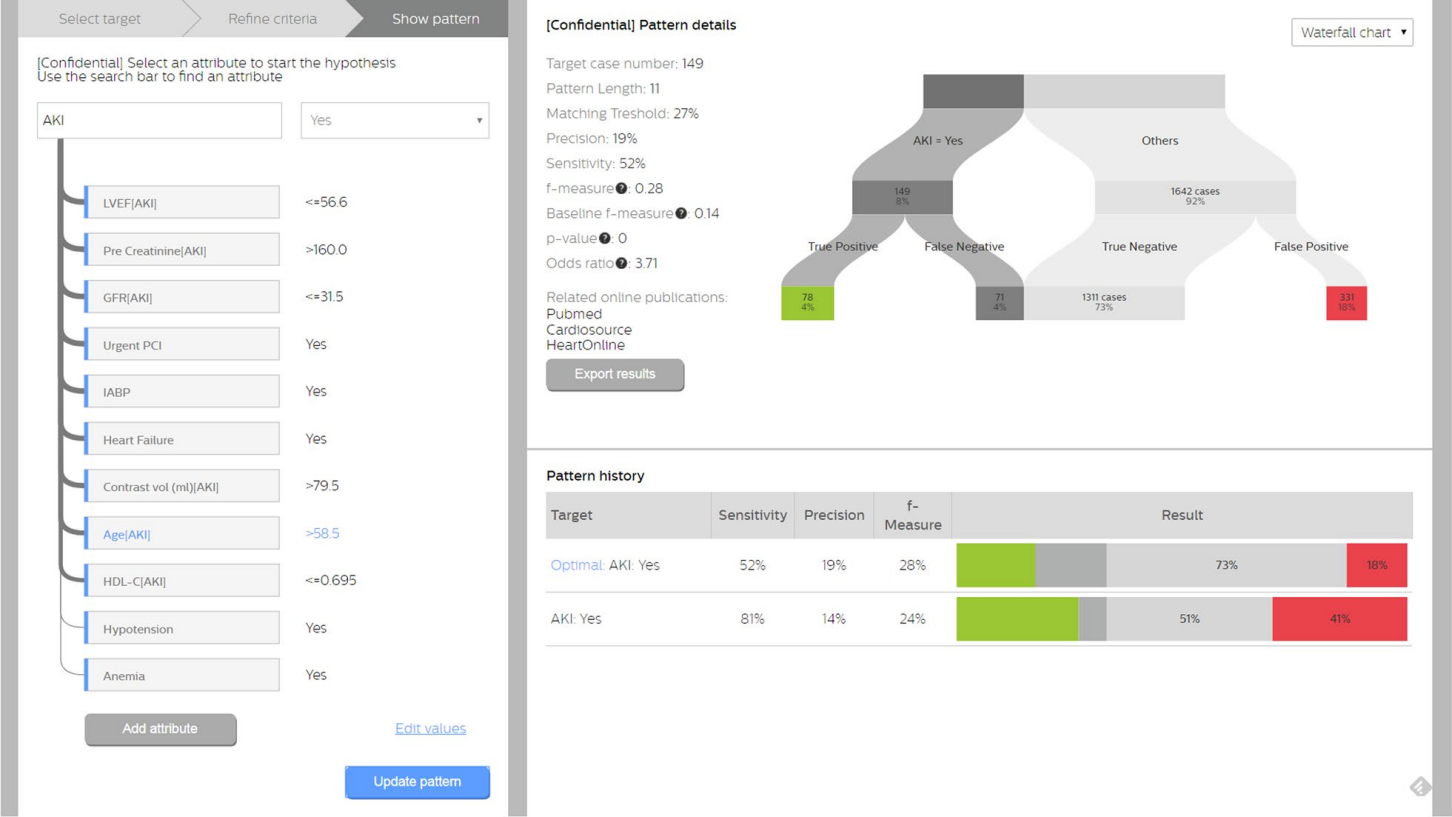
Pattern discovery based visual analytics tool using the in-mode of knowledge incorporation: age in pattern changed by clinician on-the-fly. Note all the prediction metrics are for the training data. The left panel displays the pattern where the modified variable Age [AKI] highlighted in blue illustrates the clinician’s domain knowledge incorporation (in-mode). In the pattern, the prediction target (AKI-Yes) is shown at the top. Pattern variables were connected via arcs indicating statistical significances of Chi-square test of independence [13]. A click on a variable removes an attribute (dimming the blue vertical bar). A click on “Add attribute” shows a pop-up list of variables that could be added by users. The top right panel shows the training predictive metrics of the pattern once “Update pattern” is clicked. The bottom right pattern shows the pattern history summary, where the last pattern is generated in the pre-mode. A click on “Export results” exports the current pattern for post-mode refinement

Pattern discovery was recently developed to work on incomplete noisy data for imbalanced target prediction, which was validated by our previous study [12]. The interpretable representation of pattern serves as a good basis to incorporate domain knowledge intuitively. We developed a pattern discovery based visual analytics tool and applied it on this AKI case study. We trained it on 70% consecutive patient records with three knowledge incorporation modes: (1) pre-: data-driven, (2) in-: clinician-interactive, and (3) post-: clinician-refined [11]. The first mode is purely data-driven without incorporating any knowledge (pre-mode), equivalent to the previous work [12]. In the other two modes, a physician using the visual analytics could change the variables and values on-the-fly (in-mode), and further modify the model afterwards (post-mode), respectively. To evaluate the performance of predictive modeling with knowledge incorporation, we tested and compared it with other models on the 30% consecutive patient records dated afterwards. Three modes of knowledge incorporation are enabled and elaborated below, which was integrated with the CDR (Fig. 1).

1. **Pre-mode**: We extended pattern discovery to handle numeric variables without requiring setting prior categorization rules, so that it can be used for mixed categorical and numeric data in a pure data-driven way without knowledge incorporation, serving as the baseline of knowledge incorporation. To categorize a numeric variable automatically, we employed the branching strategy in decision trees [14]. All unique values of the variable are sorted in ascending order, among which a numeric cutoff *x* is determined so that maximal information gain for the target variable is achieved by categorizing (training) data of the variable as “≤ *x*” or “ > *x*” accordingly.
2. **In-mode**: We developed the visual analytics tool, where clinician users can view and edit an existing pattern (e.g., from pre-mode) interactively through adding, removing variables, and choosing variable values according to their domain knowledge. The tool rediscovers the pattern on-the-fly and shows the updated training predictive metrics.
3. **Post-mode**: After the discovered pattern is exported, clinician users can further refine the pattern solely from their knowledge without referring to the training data, such as manually changing the numeric values in the pattern or the optimized matching ratio.

Continuous variables were reported as mean ± SD and categorical variables as percentages (%) for all participants. Normally distributed continuous variables were compared using one-way ANOVA. Four common machine learning predictive algorithms including logistic regression [15], decision trees [14], random forest [16], and Easy Ensemble [17], which were state-of-arts method handling imbalanced prediction targets, were also used for comparing the performance. In all models, the clinician user did not have access to the testing data. All three resultant models were tested on the 30% consecutive patients and compared with existing risk scores and other trained machine learning models. We evaluated the areas-under-curve (AUCs) of the receiver operating characteristics (ROC) curve, which measures the model trade-off between sensitivity and specificity. To measure the performance for imbalanced target prediction, F-score [20] considering both precision and sensitivity was reported, so was G-mean [21], the geometric mean of specificity and sensitivity.

Except AUC, all other point-specific performance metrics correspond to a certain cutoff for each model. In pattern discovery, this was auto determined by the matching threshold during training. For Mehran’s, Chen’s, Gao’s, and AGEF risk scores, we found their published thresholds yielded poor point-specific performance. Therefore, we reported their results associated with the optimal ROC points, in order not to understate their performance in case proper thresholds could be somehow obtained. For other machine learning methods except pattern discovery and Easy Ensemble, we found that imbalance showed great challenge as reported previously [12], generating trivially bad testing performance. In order not to understate their top potential performance and to stay focused on knowledge incorporation, we did random up-sampling (positive samples) and down-sampling (negative samples) to 1:1 in training for these methods and reported whichever better testing results. Other advanced techniques handling imbalance [18, 19] are beyond our scope. All analyses were performed using R (http://www.R-project.org) and Python (https://www.python.org). A *p* value of < 0.05 (two-sided) was considered statistically significant for all tests.

## Results

Among a total of 2560 patients who met the inclusion and exclusion criteria, 7.4% (N = 189) had AKI, including 4.9% (N = 126) of stage 1, 1.2% (N = 31) of stage 2 and 1.2% (N = 31) of stage 3, respectively, which is a typical imbalanced target in predictive modeling. The first 70% (N = 1791) consecutive records were used for training. The remaining 30% (N = 769) recent consecutive records were used for testing and comparisons.

**Fig. 2:**
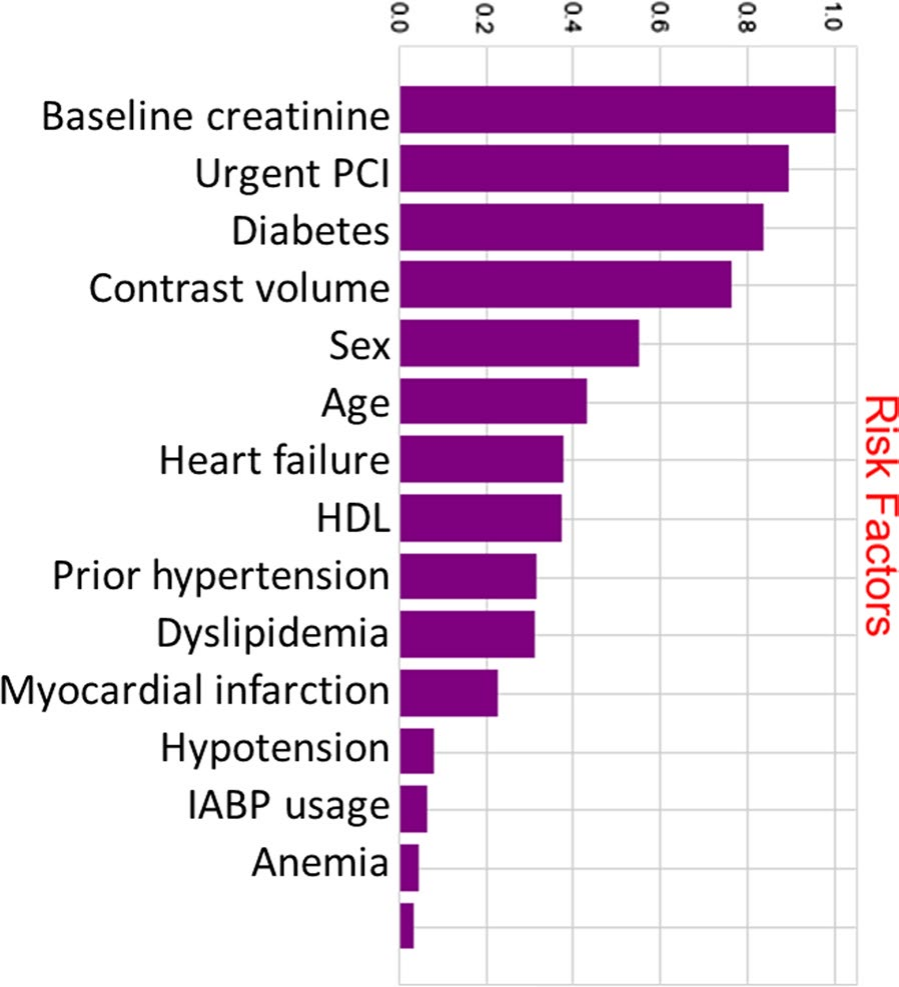
The risk factors importance from Random Forest for AKI. PCI, percutaneous coronary intervention; HDL, high density lipoprotein cholesterol; IABP, intra-aortic balloon pump; AKI, acute kidney injury

The general statistics of the 16 input variables and AKI training and testing patient records are shown in Table 1 and the risk factors’ importance from Random Forest for AKI was shown in Fig. 2. We show example categorized versions of age and left ventricular ejection fraction (LVEF) where there is no significant training–testing difference. Potential concept drift stems from the significant training–testing difference for AKI (*p* = 0.007). Reduced AKI (5.2%) in testing data may be attributed to improved procedure handling with reduced contrast volume (*p* < 0.001), increased urgent PCI (*p* = 0.019) among other factors besides fewer anemia (*p* = 0.016) patients. This consecutive testing with concept drift is more challenging than conventional cross-validation where target distribution is maintained in testing [20].

**Table 1:** Training and testing statistics of the AKI case study

	Training (N = 1791)	Testing (N = 769)	P value
Age, mean (SD), years	64.37 (11.07)	64.21 (11.00)	0.742
Age (> 60)*, n(%)	1104 (61.6%)	488 (63.5%)	0.413
Male, n (%)	1189 (66.4%)	516 (67.1%)	0.769
Anemia, n (%)	33 (1.8%)	27 (3.5%)	0.016
Diabetes, n (%)	783 (43.7%)	345 (44.8%)	0.623
Heart Failure, n (%)	127 (7.1%)	63 (8.2%)	0.372
Hypotension, n (%)	20 (1.1%)	8 (1.0%)	0.970
MI history, n (%)	127 (7.1%)	49 (6.4%)	0.566
Hypercholesterolemia, n (%)	1542 (86.1%)	683 (88.8%)	0.071
Urgent PCI, n (%)	204 (11.4%)	114 (14.8%)	0.019
Hypertension, n (%)	1251 (69.8%)	553 (71.9%)	0.612
IABP, n (%)	4 (0.5%)	12 (0.7%)	0.867
Contrast volume, mean (SD), mL	135.23 (71.17)	124.46 (63.90)	< 0.001
GFR, mean (SD), ml/min	77.76 (26.44)	82.56 (26.86)	0.248
HDL-C, mean (SD), mmol/L	1.02 (0.26)	1.02 (0.25)	0.784
Pre peak creatinine, mean (SD), μmol/L	109.78 (18.80)	106.76 (19.58)	0.558
LVEF, mean (SD), %	66.27 (11.37)	66.36 (10.96)	0.841
LVEF (≤ 45%)*, n (%)	103 (5.7%)	44 (5.7%)	0.949
AKI, n (%)	149 (8.3%)	40 (5.2%)	0.007

SD, standard deviation; MI, myocardial infarction; PCI, percutaneous coronary intervention; IABP, intra-aortic balloon pump; GFR, glomerular filtration rate; HDL-C, high density lipoprotein cholesterol; LVEF, left ventricular ejection fraction; AKI, acute kidney injury

*Categorized versions to illustrate training–testing consistency of the variables even after categorization

Using pattern discovery visual analytics, three models were generated according to the knowledge incorporation modes.

1. **Pre-mode:** the 11-variable pattern was discovered on the training data purely according to the current algorithm [11]. It reads: LVEF ≤ 56.6%, pre peak creatinine > 160 μmol/L, glomerular filtration rate (GFR) ≤  31.5 ml/min, urgent PCI = Yes, intra-aortic balloon pump (IBAP) = Yes, contrast volume > 79.5 ml, age ≤ 58.5 years old, high density lipoprotein cholesterol (HDL-C) ≤ 0.695 mmol/L, hypertension = Yes, anaemia = Yes, with the matching ratio 18%, which means a patient record has to match at least 2 out of the 11 variables to be a positive pattern match.
2. **In-mode:** based on the pre-mode pattern, the clinician user (Dr. YX Li in our author list) was free to modify pattern variables through the interface. The user changed Age from ≤ 58.5 to > 58.5 according to clinical knowledge on age as a risk factor, and did not modify other variables, because they were consistent with the clinical knowledge of the risk factors. As illustrated in Fig. 1, the re-discovered pattern maintained the same set of variable-value pairs, while the matching ratio was automatically updated to 27% (i.e., at least 3 to match).
3. **Post-mode:** Upon the in-mode pattern, the clinician user further refined Age to > 70, and contrast volume to > 100 according to experience without referring to training data. No change was made to the matching ratio.

The performance comparison results are shown in Table 2, with models of best performance highlighted in bold. Both in-mode and post-mode models with knowledge incorporation demonstrate improved AUC (0.80 and 0.82) on top of the pre-mode performance (0.77). Knowledge incorporation models demonstrated better balanced specificity and sensitivity compared to the risk scores developed from elsewhere. All four risk scores sacrificed sensitivity remarkably for specificity, resulting in compromised AUCs (0.62–0.72). Machine learning methods without proper imbalance handling were no better than existing risk models on AUCs (0.58–0.64), even though resampling was applied. The top data-driven method Easy Ensemble produced a closer AUC (0.70). Similar conclusions on F-scores and G-means demonstrate the advantage of domain knowledge incorporation with data-driven machine learning to overcome concept drift in this real AKI use case.

**Table 2:** Testing results of the three knowledge incorporation models in comparison with other risk scores and machine learning methods

Model	AUC	Sensitivity	Specificity	F-score	G-mean
(1) Pre-mode	0.77	**0.83**	0.57	0.17	0.69
(2) In-mode	0.80	0.70	0.80	0.26	**0.75**
(3) Post-mode	**0.82**	0.60	0.88	**0.32**	0.73
Mehran’s (> 7.8)	0.70	0.24	**0.94**	0.20	0.47
Chen's (≥ 13)	0.72	0.42	0.88	0.24	0.61
Gao's (> 5)	0.67	0.34	**0.94**	0.29	0.57
AGEF (≥ 0.66)	0.62	0.37	0.88	0.21	0.57
Logistic regression	0.59	0.84	0.33	0.12	0.53
Decision tree	0.58	0.61	0.55	0.12	0.58
Random forest	0.64	0.58	0.72	0.17	0.64
Easy ensemble	0.70	0.61	0.79	0.23	0.69

The evaluation metrics are defined as follows:

Specificity = TN/(TN + FP); Sensitivity = TP/(TP + FN); Precision = TP/(TP + FP); F-score = 2*Precision*Recall/(Precision + Recall) if TP > 0 and 0 if TP = 0; TP is the count of true positives, FP of false positive, TN of true negatives and FN of false negatives

AUC, areas-under-curve; AGEF, Age, Glomerular filtration rate and Ejection Fraction

## Discussion

We have reported our initial results of knowledge incorporation utilizing pattern discovery for AKI predictive modeling with data of cardiac catheterization patients in Peking University First Hospital. Our models with knowledge incorporation generated from training data have demonstrated promising predictive performance in consecutive testing data compared to existing risk models and other data-driven machine learning methods.

Similar with previous studies, existing AKI predictive models were found to have poor predictive performance when generalized into different population [8–10]. With the development of CDRs and EHR in China, more and more data generated with informatics system are available, however, challenges such as missing data or concept drift [3], increased the difficulties for using these data in real world practice. Proposed in recent work [12], a pattern was represented as a set of variable-value pairs with an optimized matching threshold, and a heuristic pattern discovery algorithm was developed. Pattern discovery has demonstrated competitive cross-validation performance on two retrospective real datasets for imbalanced target prediction. Interpretable patterns can provide insights in an intuitive way. Therefore, we developed a pattern discovery based visual analytics tool and applied it in this case study. Furthermore, our current model uses data from CDR and EHR system, which makes the model could be calculated in real time to identify high-risk patients in the future.

There are also many challenges for implanting machine learning and deep learning algorithm into clinical prediction. As described by Vapnik and Vashist [21] as ‘learning using privileged information’ paradigm, external information is actually used at the time of training to improve the incurring decision rule. So that we argue that incorporating clinical domain knowledge in an intuitive way could improve predictive models using an integrated tool with CDR, which is friendly using for physicians to collaborate with data scientists. And the results of this case study demonstrate the advantage of incorporated domain knowledge which could alleviate the challenge of concept drift compared to pure data-driven models.

This study has several limitations. First, the dataset was limited as single center, which could introduce bias and lack of generalization. A consecutively enrollment of all cases could minimize related bias, and pre-structured data input was used to deal with data missing issue. Secondly, the definition of AKI was only based on change of creatinine based on AKIN, which could underestimate the incidence of real clinical AKI, however, this definition and methods of AKI identification was used in many previous studies, which were validated and with good feasibility based on CDRs and EHR data. In future work, we will further evaluate and enhance the tool with more case studies, as well as investigate into extra variables to improve AKI prediction.

In conclusion, we developed a novel pattern-discovery-based outcome prediction tool integrated with CDR and purely using EHR data. On the case of predicting contrast related AKI, the tool showed user-friendliness by physicians, and demonstrated a competitive performance in comparison with the state-of-the-art models.

## Data Availability

The datasets of the current study are not publicly available: due to reasonable privacy and security concerns, the underlying EHR data are not easily redistributable to researchers from other centers.
